# Characterizing a photoacoustic and fluorescence imaging platform for preclinical murine longitudinal studies

**DOI:** 10.1117/1.JBO.28.3.036001

**Published:** 2023-03-07

**Authors:** Weylan R. Thompson, Hans-Peter F. Brecht, Vassili Ivanov, Anthony M. Yu, Diego S. Dumani, Dylan J. Lawrence, Stanislav Y. Emelianov, Sergey A. Ermilov

**Affiliations:** a PhotoSound Technologies, Inc., Houston, Texas, United States; b Georgia Institute of Technology, Department of Biomedical Engineering, Atlanta, Georgia, United States

**Keywords:** photoacoustic, fluorescence, mouse, tomography, medical imaging

## Abstract

**Significance:**

To effectively study preclinical animal models, medical imaging technology must be developed with a high enough resolution and sensitivity to perform anatomical, functional, and molecular assessments. Photoacoustic (PA) tomography provides high resolution and specificity, and fluorescence (FL) molecular tomography provides high sensitivity; the combination of these imaging modes will enable a wide range of research applications to be studied in small animals.

**Aim:**

We introduce and characterize a dual-modality PA and FL imaging platform using *in vivo* and phantom experiments.

**Approach:**

The imaging platform’s detection limits were characterized through phantom studies that determined the PA spatial resolution, PA sensitivity, optical spatial resolution, and FL sensitivity.

**Results:**

The system characterization yielded a PA spatial resolution of 173±17  μm in the transverse plane and 640±120  μm in the longitudinal axis, a PA sensitivity detection limit not less than that of a sample with absorption coefficient $\mu_{a}=0.258\;{\mathrm{cm}}^{-1}$, an optical spatial resolution of 70  μm in the vertical axis and 112  μm in the horizontal axis, and a FL sensitivity detection limit not <0.9  μM concentration of IR-800. The scanned animals displayed in three-dimensional renders showed high-resolution anatomical detail of organs.

**Conclusions:**

The combined PA and FL imaging system has been characterized and has demonstrated its ability to image mice *in vivo*, proving its suitability for biomedical imaging research applications.

## 1 Introduction

Photoacoustic (PA) imaging (PAI) is an emerging imaging modality that takes advantage of the optical absorption properties of tissue to generate high-resolution *in vivo* images of anatomy and exogenous chromophores.^1^ PAI works through the PA effect: pulsed light delivered to a sample is absorbed, thereby causing thermoelastic expansion and generating acoustic pressure waves.^2^^,^^3^ These acoustic waves are detected by an ultrasound transducer and reconstructed to form an image. In the near-infrared (NIR) window, oxyhemoglobin and deoxyhemoglobin have relatively high optical absorption coefficients compared to other endogenous chromophores, enabling PAI to detect and highlight blood-rich tissues *in vivo* with a spatial resolution comparable to ultrasound imaging.^4^^–^^8^ The high contrast and sensitivity of these blood-rich tissues are responsible for PAI’s suitability as a functional and anatomical imaging modality. Additionally, PA contrast agents are designed with optical absorption properties that can be exploited with multispectral (i.e., multiwavelength) PAI.^9^^–^^14^

Recent advances in PA system engineering have brought forth three-dimensional (3D) whole-body PAI as a contender for rodent, specifically mouse, studies. Compared to established preclinical imaging modalities, such as magnetic resonance imaging and nuclear imaging (e.g., positron emission tomography, single-photon emission computed tomography), PAI has several advantages, including higher temporal resolution, higher spatial resolution, detection specificity of chromophores, non-ionizing radiation imaging, and relatively low cost.^15^ The development and characterization of rodent models, as well as their use in the preclinical phase of drug and medical device research, promotes *in vivo* whole-body imaging modalities.^16^^–^^18^ PAI has demonstrated 0.2 to 0.5 mm deep-tissue spatial resolution in high-fidelity, 3D, whole-body mouse images.^19^ These image volumes can provide anatomical maps of skin, vasculature, and blood-rich tissue. Through the use of spectral unmixing algorithms, multispectral PA images can be used to construct physiological maps of blood,^20^^,^^21^ lipid content,^22^ blood hypoxia,^23^ and perfusion.^24^^,^^25^

The PAI is also an excellent tool for quantitative molecular imaging due to the wide variety and commercial availability of molecular sensors for preclinical studies (i.e., contrast agents, nanoparticles, and fluorophores). Fluorophores generate a near-simultaneous fluorescence (FL) emission and PA response when excited by nanosecond-range laser pulses;^26^^,^^27^ thus, PAI and FL imaging (FLI) have a natural synergy as detection modalities.

FLI is a widely used optical imaging modality that has high molecular sensitivity to fluorophores. In comparison to PAI, FLI is generally considered to have a higher molecular sensitivity since it is unburdened by background noise generated from endogenous chromophores.^28^^,^^29^ FLI has submillimeter resolution at superficial tissue (<2  mm tissue depth), with deeper tissue having significantly worse spatial resolution (<5  mm resolution), prohibiting accurate, volumetric, and quantitative imaging of localized molecular sensor concentrations *in vivo* as compared to PAI.^30^ This imaging depth and spatial resolution weakness of FLI can be countered by the addition of a PAI mode, with both modes complimenting one another through high sensitivity (FLI) and high-resolution (PAI) imaging of fluorophores.

Here, we introduce a preclinical imaging system, the TriTom, that integrates PA and FL imaging to capitalize on the strengths of both modalities and mitigate the disadvantages of either technique. Compared to other combined PAI and FLI systems,^31^^–^^33^ our imaging platform achieves coregistered PA tomography (PAT) and FL molecular tomography (FMT) to enable 3D volumetric imaging with high-resolution and high molecular sensitivity. Additionally, the TriTom acquires whole-body *in vivo* azimuthal PA frame data and planar optical data simultaneously, allowing for the temporal co-registration of the reconstructed PAT and FMT volumes. In this work, we characterize the PA spatial resolution, PA sensitivity, PA spectral accuracy, optical spatial resolution, and FL sensitivity of the TriTom imaging platform and demonstrate anatomical PAI with an *in vivo* mouse experiment.

## 2 Materials and Methods

### 2.1 Dual-Modality Preclinical Imaging System

**Fig. 1:**
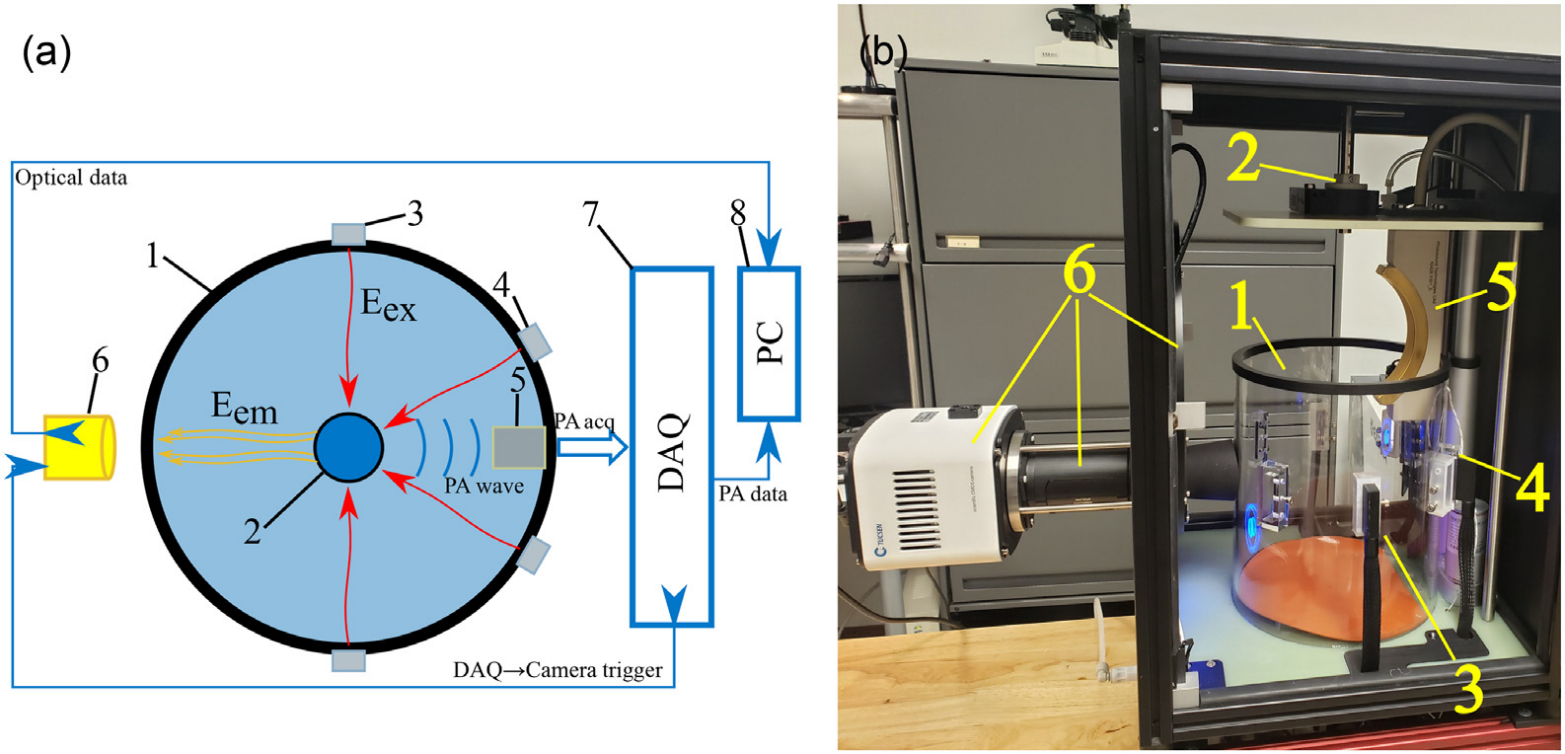
Diagram of the (a) TriTom imaging chamber layout and (b) assembled TriTom imaging chamber. Quartz glass water tank (1), rotational stepper motor and sample/animal mounting point (2), orthogonal excitation termini slots (3), epi-excitation termini slots (4), PA detector array (5), FL imaging camera, adjustable objective, and optical filter wheel (6), data acquisition board (7), and Windows 10 PC (8). Laser excitation ($E_{\mathrm{ex}}$) light is shown as a wavy red arrow, FL emission ($E_{\mathrm{em}}$) light is shown as a wavy yellow arrow, a PA wave is shown as a curved blue line, and the direction of data acquisition flow and triggers are shown as blue arrows.

The TriTom is a dual-modality imaging platform that includes both a PA detector array and an FL imaging scientific complementary metal-oxide-semiconductor (sCMOS) camera. A top-down diagram [Fig. 1(a)] of the imaging chamber and data acquisition process details the system’s key components. The cylindrical imaging chamber (1) has an inner diameter of 190 mm and wall thickness of 5 mm made of glass quartz. The rotational stepper motor (2) (K10CR1, Thorlabs, Newton, New Jersey, United States) has a hollow shaft placed through it that acts as the mount point for samples. The motor’s maximum speed of rotation is 10  deg/sec and determines the time of a 360-deg TriTom scan as 36 s. The orthogonal excitation termini slots (3), orthogonal to the scanning plane of the PA array, are positioned on the outer circumference of the cylindrical imaging chamber. The epi-excitation termini slots (4) are positioned 60 deg from the orthogonal excitation termini slots towards the PA detector array. All excitation termini are vertically aligned with the centermost element of the PA array. Randomized fiber bundle outputs, of lightbar surface dimensions $1\times 30\;{\mathrm{mm}}^{2}$, are slotted into the excitation termini slots (3, 4). The fiber bundle inputs are routed to the laser’s (PhotoSonus, Ekspla, Vilnius, Lithuania)^34^ optical parametric oscillator (OPO) output. The laser’s OPO output had a wavelength range from 670 to 1064 nm and was pulsed at 10 Hz with a 5-ns pulse duration.

The acoustic waves induced by laser excitation are detected by a curve-linear 96-element PA transducer detector array (5) (Imasonic SAS, Voray sur l’Ognon, France). The piezoelectric transducer elements ($1.3\times 1.3\;{\mathrm{mm}}^{2}$) were positioned on the centerline of the curved array with inter-element spacing of 0.1 mm. The center frequency of the array was 6  MHz±10% (at −6  dB) with bandwidth ≥55%. The array was positioned vertically with the centermost element aligned to the rotational axis, which was spaced 65 mm from the array’s center element. For FL imaging, an sCMOS camera (2048−2040  pixels) (6) (Dhyana 400D, Tucsen, Fuzhou, China) was positioned outside of the imaging chamber with two optical components between it and the chamber’s circumference. The camera had a quantum efficiency (QE) of 45% at 800 nm, but had an operational (>20% QE) wavelength range of 400 to 900 nm. The camera’s readout noise was 2.5e- for its high dynamic range mode (16-bit). A combination objective lens and aperture (Contrastech, Hangzhou, China) with a focal length of 50 mm and f/2.0 to 22 was placed directly after the camera. A filter wheel (Starlight Xpress, Berkshire, United Kingdom) was mounted inside the imaging module that allows the camera’s acquisition of FL images, with an appropriate optical filter selected. The described configuration resulted in an FL imaging optical detection channel with a $40\times 40\;{\mathrm{mm}}^{2}$ field-of-view (FoV) plane focused at the rotational axis.

The signals from the PA detector are captured by a 128-parallel channel data acquisition board (DAQ) (7) (DAQ128, PhotoSound Technologies, Houston, Texas, United States) capable of acquiring 4096 samples per channel at a sampling rate of 40 MHz. The DAQ had a gain range of 46 to 91 dB (40 dB preamplifier, 6 to 51 dB programmable gain) and was optically triggered by a single fiber routed from the fiber bundle input. Captured signals were filtered through a 10 MHz low-pass filter and transferred to a Windows 10 PC (8) via a USB 3.0 connection, along with data from the stepper motor and camera, for processing. This combination of collected data was then used to reconstruct PA and FL volumes.

### 2.2 Characterization of the TriTom Imaging Platform

Three phantoms were prepared to characterize the spatial resolution, sensitivity, and multispectral imaging capabilities of the PA imaging modality. Two phantoms were prepared to evaluate the optical spatial resolution and sensitivity of the FL imaging modality. The laser’s OPO output energy was set to 80  mJ/pulse for all wavelengths used in phantom experiments, to minimize variations in fluence for each scan. The TriTom imaging chamber was filled with degassed deionized (DI) water at a temperature of 25.0°C±0.5°C for all phantom scans.

#### 2.2.1 Photoacoustic spatial resolution phantom

**Fig. 2:**
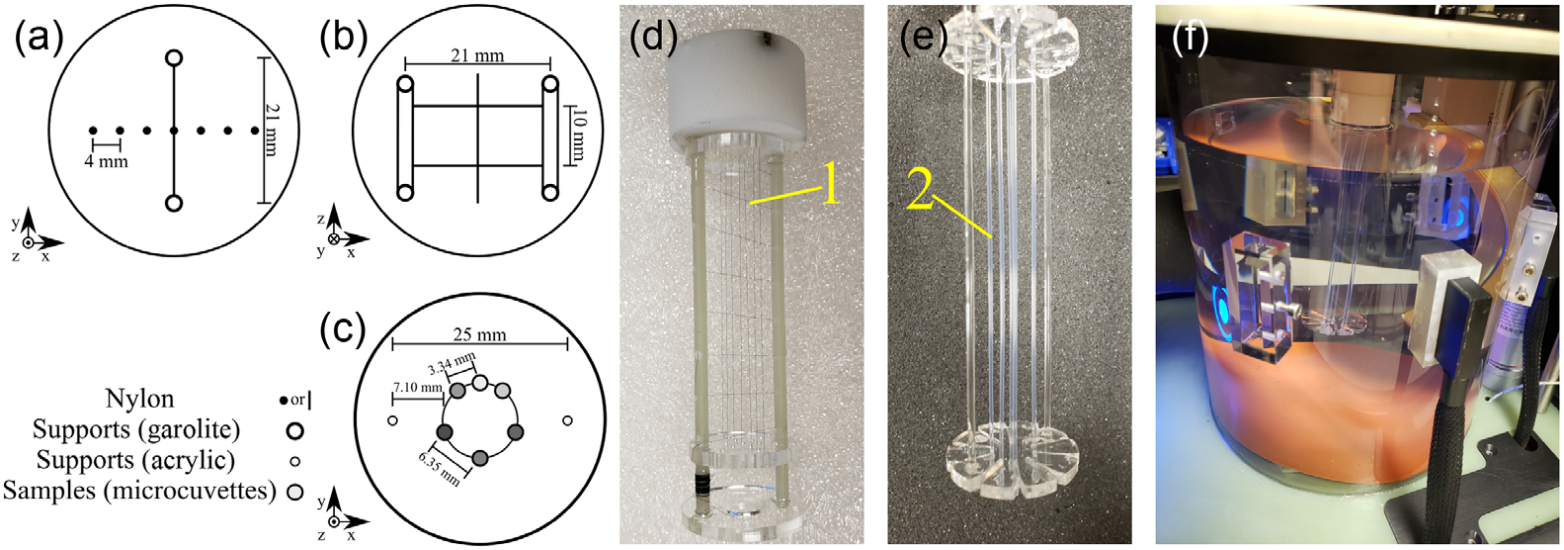
Diagrams of the PA spatial resolution phantom’s (a) top view and (b) side view, as well as the PA sensitivity phantom’s top view (c); the multispectral phantom had a similar configuration to the PA sensitivity phantom. Photos of (d) the PA spatial resolution phantom, (e) PA sensitivity phantom, and (f) a phantom installed in the system’s imaging chamber. Key components: Ø50  μm monofilament matrix (1) and sample microcuvettes (2).

The PA spatial resolution phantom [Figs. 2(a), 2(b), and 2(d)] was assembled by threading black polyamide 6,10, Ø50  μm, monofilament strings through a resolution phantom matrix. A total of fourteen strings were used to construct the PA spatial resolution phantom with seven threaded vertically (spaced 4 mm apart) and seven threaded horizontally (spaced 10 mm apart).

**Fig. 3:**
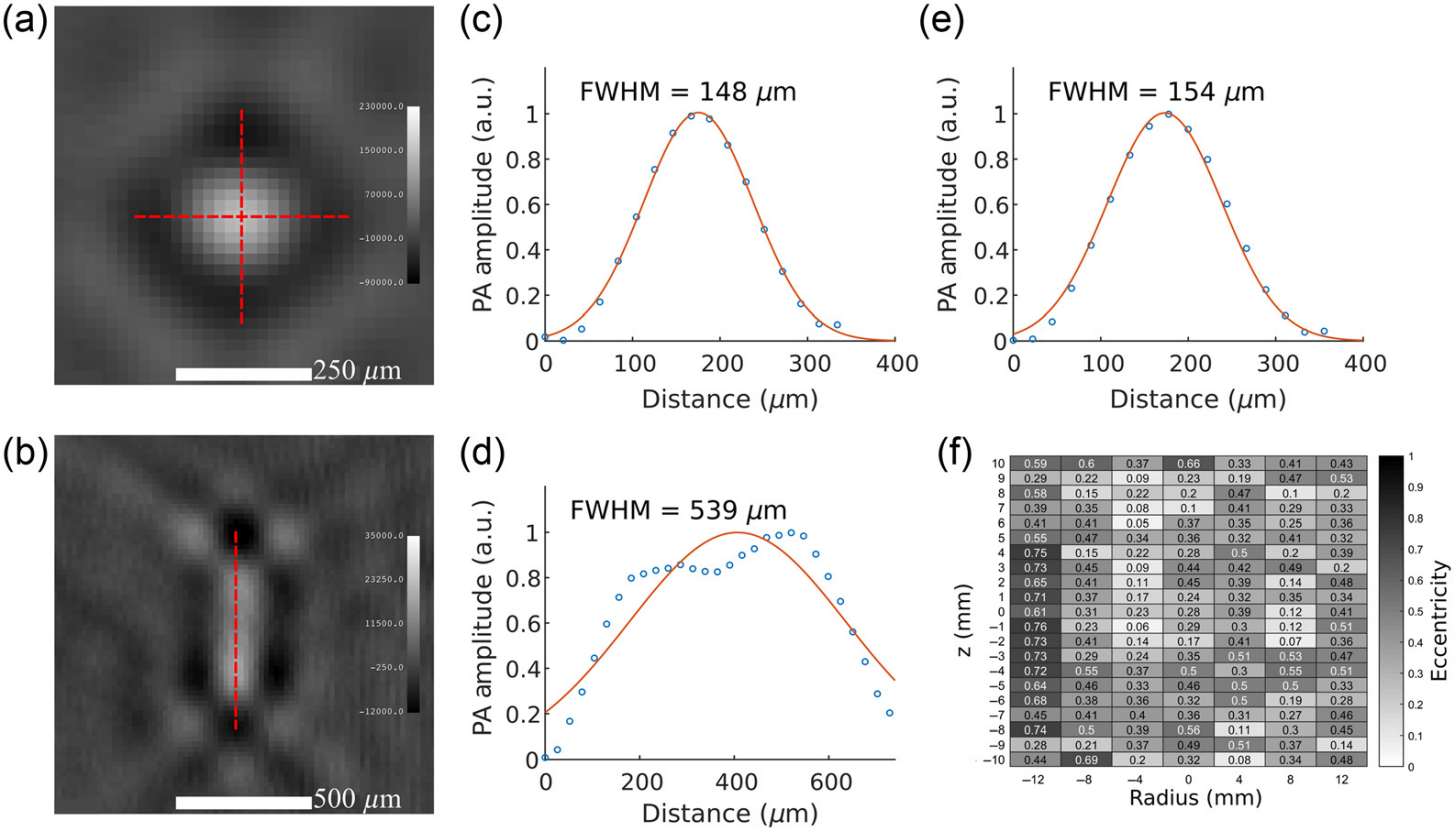
Red dashed line profile on a transverse (a) and longitudinal (b) slice of the spatial resolution phantom monofilaments. (c)–(d) Sample Gaussian fits over measured line profiles for the x-axis (c), y-axis (e), and z-axis (d). (f) Heatmap of eccentricity values from the ellipse formed by the x-axis and y-axis FWHM values.

The assembled phantom was scanned using the system’s PA modality at an excitation wavelength of 800 nm. The PA spatial resolution of the system was determined by extracting line profiles of vertical and horizontal monofilaments from the reconstructed volume with a voxel size of 0.02 mm (Fig. 3). Gaussian distributions were fitted over the line profile data and the full width at half max (FWHM) was calculated as the spatial resolution metric. To determine the transverse (axial) resolution, radial and tangential line profiles were measured from each vertical monofilament between a ±10  mm vertical window with 1-mm steps [Figs. 3(a), 3(c), and 3(e)]. Since the transverse PA spatial resolution would be the average of the radial and tangential FWHM, an elliptical eccentricity chart was calculated using these two FWHM values for each vertical monofilament line profile measurement [Fig. 3(f)]. Similarly, the vertical resolution was measured by taking the vertical line profile of the two visible horizontal monofilaments between a ±10  mm horizontal window with 1-mm steps [Figs. 3(b) and 3(d)].

#### 2.2.2 Photoacoustic sensitivity phantom

The PA sensitivity phantom [Figs. 2(c), 2(e), and 2(f)] was constructed using six polytetrafluoroethylene (PTFE) microcuvettes (Zeus, Orangeburg, South Carolina, United States) with an inner diameter of Ø0.81±0.03  mm and wall thickness of 0.05±0.03  mm. Five PTFE microcuvettes were filled with increasing concentrations of a stable and well-characterized PA chromophore: ${\mathrm{CuSO}}_{4}{\cdot 5H}_{2}O$^35^ (1.9, 4.9, 10.3, 24.7, and 49.6 mM, respectively) and the remaining microcuvette was filled with DI water (specific resistivity >18.2  MΩcm) as a control. The PTFE microcuvettes were arranged in the sample holder in a circular pattern with 4-mm spacing between samples and 5.4-mm separation from the center of the phantom holder. The sensitivity phantom was then mounted in the TriTom imaging chamber and scanned at 800 nm. Additionally, the phantom was imaged in a scattering environment consisting of a latex condom filled with skim milk,^36^ which had a radius of roughly 17 mm from the phantom’s center making the closest distance between the sample microcuvette and scattering barrier 11.4 mm.

Each scan was then reconstructed into a 3D PA volume and analyzed. For each sample, the microcuvette was manually segmented and the mean target inclusion (i.e., the average intensity of the segmented volume) was calculated. We define the lower limit of detectability (LLOD) (i.e., limit of quantitation^37^) of the PA mode as a sample’s target inclusion having a contrast-to-noise ratio (CNR) value greater than two^38^ with CNR defined in the following equation:^39^

$$\mathrm{CNR}=\frac{A_{\text{sample}}-A_{\text{background}}}{\sigma_{\text{background}}},$$ (1)

where A is the mean target inclusion and σ is the standard deviation. The PA sensitivity phantom’s samples were segmented within the vertical boundary of ±10  mm, then averaged. The background noise was segmented, with mean and standard deviation values calculated on the segmentation. The CNR of each concentration sample was calculated according to Eq. (1) for both scattering and non-scattering environments.

#### 2.2.3 Photoacoustic multispectral phantom

To characterize the multispectral PA capabilities of the TriTom, three PAI contrast agents, with distinct optical absorbance spectra, were prepared with concentrations adjusted to match an optical density (OD) peak of $1\;{\mathrm{cm}}^{-1}$ per sample at their peak optical absorption wavelengths: ${\mathrm{CuSO}}_{4}{\cdot 5H}_{2}O$ (99.2 mM, 810 nm), indocyanine green (ICG) (26.5  μM, 780 nm), ${\mathrm{NiSO}}_{4}\cdot 6H_{2}O$ (13.6 M, 720 nm), and a control sample of DI water. The samples’ optical spectra were measured using a spectrophotometer (GENESYS 30, Thermo Fisher Scientific, Waltham, Massachusetts, United States). A phantom was assembled by filling PTFE microcuvettes with each sample and placing them into a sample holder, then mounting the holder into the TriTom. Multispectral PA scans were then acquired from 690 to 890 nm at intervals of 10 nm with an average laser energy of 80 mJ per pulse for each wavelength scan.

A PA spectrum for each sample was extracted from the reconstructed volumes of the multispectral phantom. Using the 800-nm volume, the sample microcuvettes’ areas were identified and segmented, from which the mean target inclusion of each sample across all wavelength volumes was calculated. The mean target inclusions were background subtracted to produce PA spectra for the samples. The resulting PA spectra were normalized to the ${\mathrm{CuSO}}_{4}{\cdot 5H}_{2}O$ spectrophotometer data to evaluate the PA spectral accuracy of the system without considering the reconstruction algorithm’s lack of physical corrections.

#### 2.2.4 Optical spatial resolution phantom

**Fig. 4:**
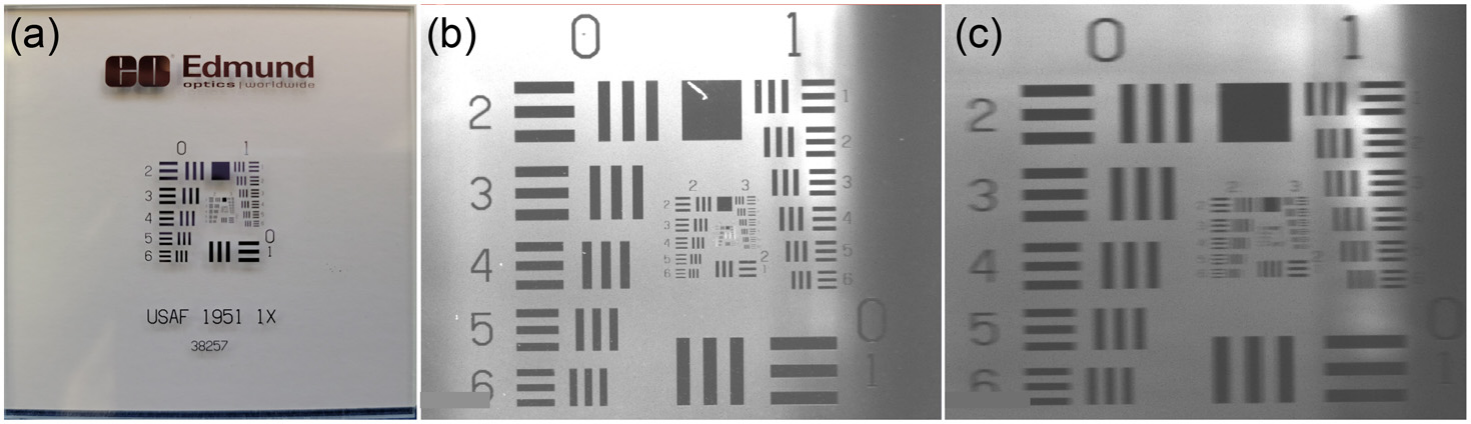
Images of the optical resolution phantom: (a) a 1951 USAF resolution test chart outside of the imaging chamber, (b) inside the imaging chamber with air, and (c) inside the imaging chamber filled with DI water.

The optical spatial resolution phantom used was a 1951 USAF resolution chart (Edmund Optics, Barrington, New Jersey, United States) [Fig. 4(a)], a standard in optical spatial resolution measurement.^40^ The phantom was mounted onto the TriTom’s hollow shaft perpendicular to the imaging axis, and the objective was manually adjusted to focus at the phantom’s plane with a fully open aperture and filter wheel set to open (no optical filters). The camera’s vertical and horizontal optical spatial resolution was measured both in air [Fig. 4(b)] and with the TriTom imaging chamber filled with DI water [Fig. 4(c)].

Vertical and horizontal line profiles were taken from the elements of the chart. If the three distinct peaks of each element were detected in its line profile, then the element is considered to be resolvable (Fig. S1 in the Supplementary Material). The optical resolution was determined to be the smallest resolvable element of the chart. The line pairs per mm values associated with each element were converted to microns, to be consistent with the reported PA spatial resolution.

#### 2.2.5 Optical fluorescence sensitivity phantom

Multiple concentrations (28.0, 13.7, 6.0, 2.6, and 0.9  μM) of IR-800 were prepared to assess the FLI sensitivity of the TriTom. The IR-800 samples were filled into PTFE microcuvettes and imaged using an IVIS Spectrum (PerkinElmer, Waltham, Massachusetts, United States) and a TriTom, with the IVIS Spectrum acting as a validation reference point for FLI sensitivity.

For the IVIS Spectrum FLI scans, the sample microcuvettes were laid out with roughly 5-cm spacing between one another. The IVIS Spectrum was set to capture with an exposure time of 200 ms, an excitation short-pass filter of 745 nm, and an emission long-pass filter of 800 nm.

The microcuvettes were then transferred to the TriTom sample holder and mounted into the imaging chamber. A band-pass 814 to 851 nm optical filter (Semrock, Rochester, New York, United States) was set to filter out the 780-nm excitation laser light and allow the IR-800 samples’ FL emission to be detected by the camera. The camera’s exposure time was set to 25 ms and dual PA and FL scan was taken. The PA and FL data were reconstructed into volumes with our standard parameters for analysis. A comparison of four image datasets was performed to assess the sensitivity of each method: IVIS Spectrum FLI, TriTom FLI, TriTom FMT, and TriTom PAT. The imaging methods had their samples segmented and processed to calculate CNR values.

### 2.3 Animal Studies

To demonstrate the *in vivo* imaging capabilities of the TriTom, three mice were imaged using TriTom instruments at two research institutions. The first and second animals’ experimental protocol was approved by the Institutional Animal Care and Use Committee at the Georgia Institute of Technology (GIT). The third animal’s study was approved by the Ethics Committee of the Saratov State University (SSU). All mice were anesthetized with isoflurane gas and imaged in a TriTom with water temperature at 37.0°C±0.5°C (Fig. S2 in the Supplementary Material).

The animals imaged at GIT were 6-week-old BALB/c nude mice (Charles River Laboratories, Wilmington, Massachusetts, United States) that had been injected with a 50-μl dose of 50  μg/ml glycol-chitosan-coated gold nanospheres (GC-AuNPs) mixed with 50  μ/ml ICG (Sigma-Aldrich, St. Louis, Missouri, United States) prior to imaging. The first mouse was imaged 6-hr post injection while the second mouse was imaged 24-hr post injection. The GC-AuNP and ICG mixture was administered as a single bolus injection subcutaneously to the right 4’th mammary fat pad for both animals. This injection site was chosen because it is not close to highly optically absorbent blood-rich organs. The purpose of the study was to track the regional clearance of the injected contrast agent *in vivo*. Additionally, a PTFE microcuvette filled with copper sulfate ($\mathrm{OD}=1.2\;{\mathrm{cm}}^{-1}$ at 800 nm) was taped to the back of the mice as a PA fiducial. During imaging, the animals were anesthetized with 2% isoflurane mixed with oxygen, secured in the mouse restrainer, and mounted in the TriTom imaging chamber. PAI scans were then acquired at 532 and 710 nm.

The first mouse was excited with a wavelength of 532 nm to highlight superficial structures of the mouse as well as being close to an absorption peak of the GC-AuNPs (Fig. S3 in the Supplementary Material). Similarly, 710 nm was close to an absorption peak of the GC-AuNPs and would also highlight deoxygenated hemoglobin (Hb) filled organs, due to Hb’s relatively high optical absorption with respect to oxygenated hemoglobin (${\mathrm{HbO}}_{2}$).

The second mouse was excited with a wavelength of 770 nm to induce FL emission from the ICG component of the dye. A band-pass optical filter 814 to 851 nm was used to filter out the laser excitation light and pass the ICG FL emission to the optical detector. The IVIS Lumina II In Vivo Imaging System (Caliper Life Sciences, Hopkinton, Massachusetts, United States) was used in combination with the IVIS Acquisition Software for an FL imaging comparison. FL images were acquired targeting ICG with a 745-nm excitation filter and an 840-nm emission filter with a 6.5-cm field of view. During data collection, the mouse was anesthetized with the Visualsonics Inc. Anesthesia System (2% isoflurane mixed with oxygen) integrated into the IVIS imaging system via a nose cone.

For the third *in vivo* imaging study, a female BALB/c mouse was imaged in the TriTom system at SSU. The mouse was anesthetized using 3% isoflurane mixed with air and the hair covering the imaging area was trimmed and removed with depilatory cream. The animal was then mounted in the mouse restrainer and transferred to the TriTom for imaging. A single PAI scan was acquired at 1064 nm with an average laser energy of 380  mJ/pulse to generate deep anatomy images of ${\mathrm{HbO}}_{2}$-rich structures.

### 2.4 Reconstruction and Visualization

#### 2.4.1 Photoacoustic reconstruction

The PAT reconstruction employed a graphics processing unit (GPU) accelerated filtered back-projection (FBP) algorithm programmed in MATLAB 2017b,^41^ with the GPU acceleration process of the code utilizing compute unified device architecture (CUDA).

A running average was applied to the PA signals to reduce frame-by-frame background noise as well as a sixth-order Butterworth low-pass filter at 8 MHz to reduce higher frequency noise outside of the PA detector’s effective frequency range. The rotational stage motor’s angle log was asynchronous with the PA data acquisition. Angular data was interpolated for each scan’s duration and each PA frame data was assigned an angle from this interpolation based on its timestamp of acquisition. Laser light factors, output laser energy, and optical water absorption were corrected for during the reconstruction, as was the signal amplification set by the variable gain. The electrical impulse response of the system, animal respiration, speed of sound variations in heterogenous tissue, and PA probe manufacturing tolerances were not accounted for in the reconstruction.

The PA spatial resolution phantom was reconstructed with a voxel size of $0.02\times 0.02\times 0.02\;{\mathrm{mm}}^{3}$ to have a high-resolution (HR) volume for measuring the TriTom’s PA spatial resolution. All other volumes were reconstructed with our standard resolution (SR) PA reconstruction parameters, voxel size $0.1\times 0.1\times 0.1\;{\mathrm{mm}}^{3}$, to match the vertical fiber bundle output lightbar dimension. The PA reconstruction algorithm could reconstruct a 2048 sample, 360 frames, 96 channel PA dataset, using our standard PA reconstruction parameters, in 19 seconds with an RTX 3080 GPU (NVIDIA, Santa Clara, California, United States).

#### 2.4.2 Fluorescence reconstruction

The FMT reconstruction used a two-dimensional (2D) x-ray transform algorithm to stack transverse planes into a 3D FL volume.^42^ This algorithm is implemented in MATLAB 2017b and utilizes multicore processing.

Because the acquisition of PA and FL data is synchronous, the same interpolated motor data from the PA reconstruction procedure was used to map the FL frame angles. Our standard reconstruction parameters for FL reconstructions were voxel size 0.1 mm and volume $40\times 40\times 40\;{\mathrm{mm}}^{3}$, to match the camera’s FoV. For an acquisition of image resolution 1024×1020 and 360 frames using our standard FL reconstruction parameters, a reconstruction took 80 seconds with a Ryzen 7 3700X CPU (AMD, Santa Clara, California, United States).

#### 2.4.3 Visualization

The PA and FL images were loaded into 3D Slicer^43^ for visualization and image processing. For 3D visualizations, color and opacity mappings were configured manually for each volume to optimize the visibility of specific anatomical structures. These alterations were applied only to the displayed images. All image analysis or quantification was implemented using the unaltered reconstructed volumes. In the case of visualizing 2D FLI, the images were overlaid on photographs for anatomical registration, with the FL opacity with respect to the photographs being 100% for values with a CNR>0. All image segmentations were performed using 3D Slicer’s segment editor module^44^ and the segment editor extra effects extension package. Further calculations were performed in either Python (3.7.6) or MATLAB (2017b).

#### 2.4.4 Statistical analysis

Cross-sectional line profiles were extracted using image processing software (3D Slicer) and exported. The exported data were loaded into MATLAB and fitted with 1D Gaussians for spatial resolution (PA and optical) calculations. The standard deviation of the generated Gaussians were used to compute the FWHM.

The linearity of datasets was evaluated using a linear regression model from MATLAB. The y-intercept was fixed at the origin for the linear models. The 95% confidence intervals of the model were also shown.

The FL CNR maps were calculated using Eq. (1), with the target inclusion defined as the local injection site and the background defined as the pixels inside the mouse boundary (Fig. S4 in the Supplementary Material). The maps were visualized with a CNR≥2 cutoff to match our criteria of LLOD. The maps with a CNR≥0 cutoff are shown in Fig. S5 in the Supplementary Material.

## 3 Results

### 3.1 Photoacoustic Spatial Resolution

**Fig. 5:**
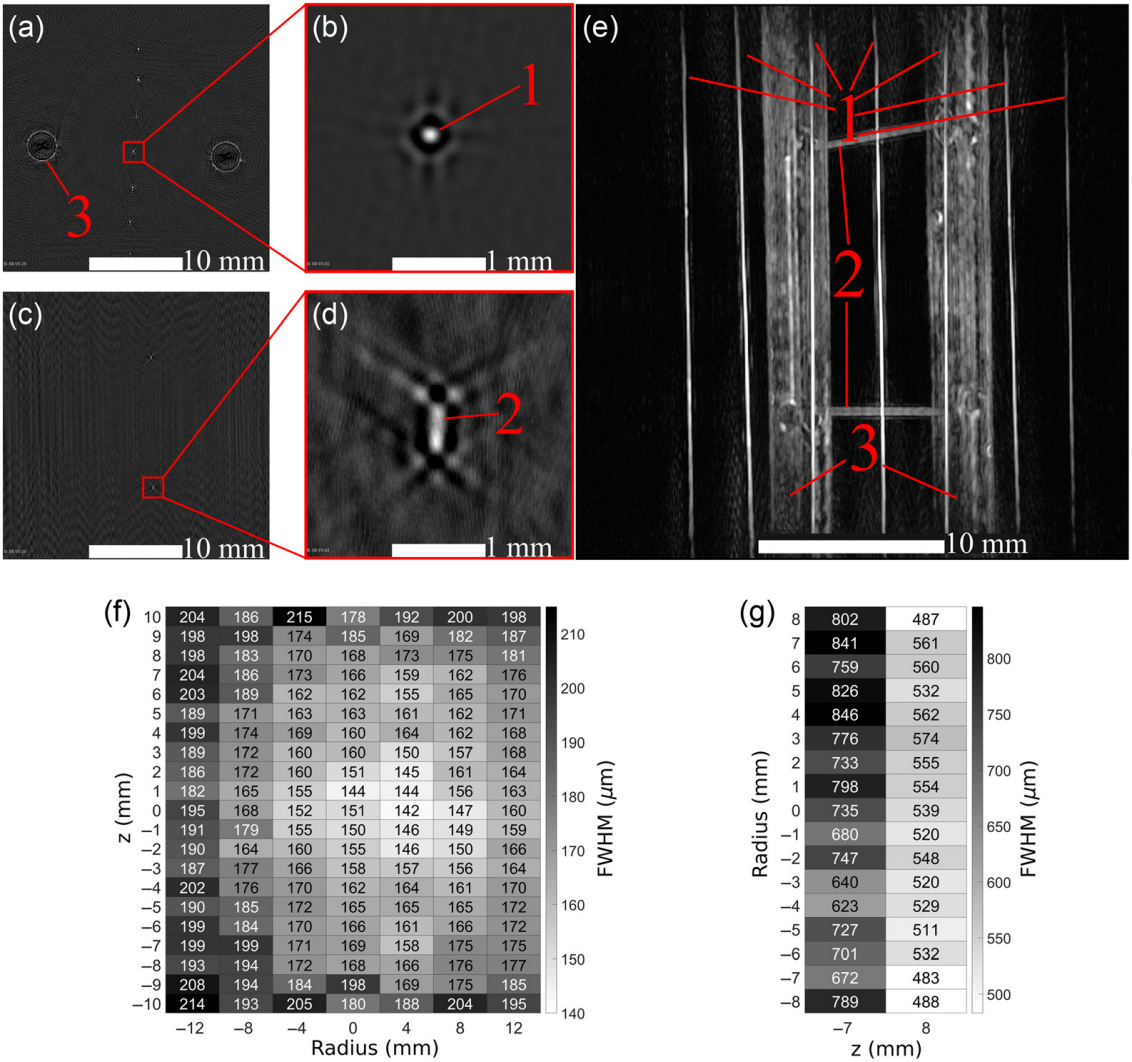
(a) The central transverse slice ($30\times 30\;{\mathrm{mm}}^{2}$) at voxel size 0.10 mm of the phantom’s vertical monofilaments, (b) a zoomed slice ($3\times 3\;{\mathrm{mm}}^{2}$) at voxel size 0.02 mm of a vertical monofilament (1). (c) A longitudinal slice ($30\times 30\;{\mathrm{mm}}^{2}$) at voxel size 0.10 mm of the phantom’s horizontal monofilaments, (d) a zoomed slice ($3\times 3\;{\mathrm{mm}}^{2}$) at voxel size 0.02 mm of a horizontal monofilament (2). (e) A 3D MIP rendering of the phantom’s 0.10-mm voxel reconstruction with the vertical monofilaments (1), horizontal monofilaments (2), and phantom support rods (3) visible. (f) A heatmap of vertical monofilaments’ average transverse FWHM between ±10  mm along the z-axis (height). (g) A heatmap of the horizontal monofilaments’, the two visible in a height range of 30 mm, longitudinal FWHM between ±10  mm along the radial direction.

PA reconstructions of the spatial resolution phantom are shown in Fig. 5. All seven vertical monofilaments were detected in the resolution phantom’s SR transverse reconstruction slice [Fig. 5(a)]; similarly, two of the horizontal monofilaments were detected in the SR longitudinal reconstruction slice [Fig. 5(c)], albeit with lower sensitivity. The HR reconstruction slices [Fig. 5(b) and 5(d)] show a 10× magnified view of a single monofilament. The entire volume was rendered as a maximum intensity projection (MIP) in 3D with a greyscale colormap [Fig. 5(e)].

Comparing the HR slices of the vertical [Fig. 5(b)] and horizontal [Fig. 5(d)] monofilaments, the transverse slice’s monofilament has an expected circular shape while the longitudinal slice’s monofilament is an oblong, dual-peak ellipse. This oblong shape can be attributed to the mechanical tolerance of the PA detector which was not accounted for in the reconstruction. As a result, the TriTom has a superior PA transverse resolution compared to its PA longitudinal resolution.

The FWHM values determined from the monofilaments’ transverse and longitudinal measurements were plotted as heatmaps [Figs. 5(f) and 5(g)] with dimensions of (radius, height). The transverse spatial resolution of the system ranges between 142 and 215  μm dependent on the radial distance from the PA detector’s focal point, with the best resolution values towards the image’s origin. While minor, the minimum spatial resolution at (4, 0) is offset from the expected (0, 0). This offset is likely due to uneven light distribution that can be corrected by optimizing the light delivery in the TriTom imaging chamber. The longitudinal spatial resolution has a larger range than the transverse resolution, between 487 and 846  μm. Continuing from the point that the horizontal monofilament reconstructions have two amplitude peaks, the line profile and FWHM measurement method for spatial resolution determination does not account for non-single peak Gaussian fits, and, therefore, will produce high estimates for spatial resolution. The reported transverse and longitudinal spatial resolution of the system is the average of the heatmap data: 173±17  μm for the transverse plane and 640±120  μm for the longitudinal dimension.

### 3.2 Photoacoustic Sensitivity

**Fig. 6:**
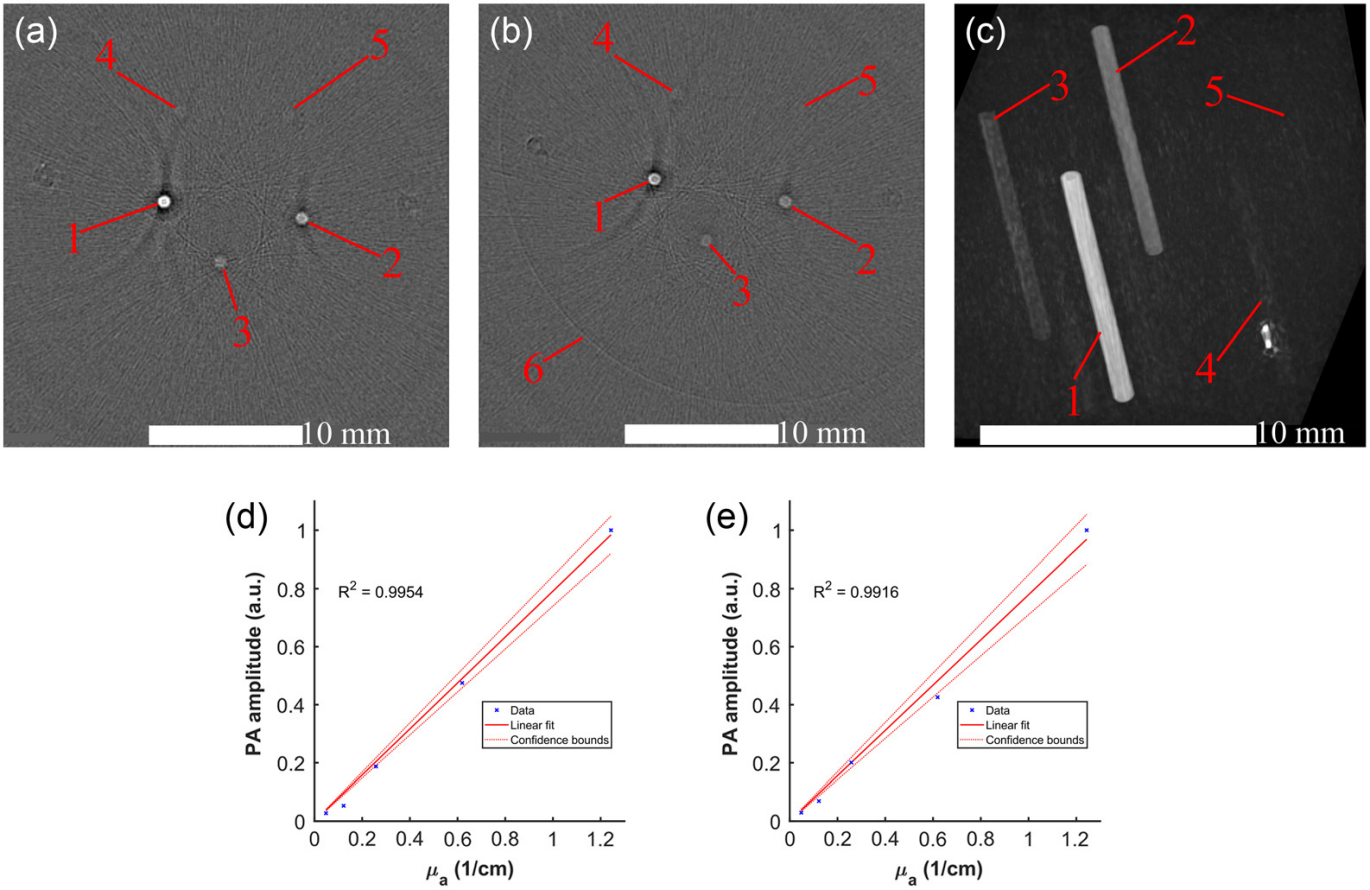
Transverse slices of the (a) non-scattering and (b) scattering PA sensitivity phantoms. (c) 3D MIP render of the non-scattering PA sensitivity phantom’s PAT volume. Linear regression fits of the PA sensitivity as a function of sample concentration for the (d) non-scattering and (e) scattering PA sensitivity phantoms.

The reconstruction of the PA sensitivity phantom, constructed with samples of copper sulfate at varying concentrations, is shown in Fig. 6. All sample concentration microcuvettes are detected above the noise floor in both the non-scattering [Fig. 6(a)] and scattering [Fig. 6(b)] environments.

The linear correlation analysis of PA response and copper sulfate concentration is shown in [Figs. 6(d) and 6(e)] for the non-scattering and scattering phantoms, respectively. The resulting r-squared values for the non-scattering (0.9954) and scattering (0.9916) cases indicate a good linear correlation between PA sensitivity and concentration. This linear relationship demonstrates the ability to reliably quantify sample concentrations from a PA amplitude analysis with the TriTom.

The CNR analysis of the sample microcuvettes is shown in Table 1 for both the non-scattering and scattering phantoms. The two lowest concentrations of ${\mathrm{CuSO}}_{4}\cdot 5H_{2}O$ (1.9 and 4.9 mM) have CNR<2 for both the non-scattering and scattering environments. This indicates that the combination of the TriTom’s PA mode hardware and the reconstruction algorithm led to a LLOD for absorption coefficients ($\mu_{a}$) between 0.122 and $0.258\;{\mathrm{cm}}^{1}$.

**Table 1:** CNR of ${\mathrm{CuSO}}_{4}\cdot 5H_{2}O$ samples’ PA reconstructions with and without a scattering environment.

Concentration (mM)	Optical absorption $\mu_{a}$ (${\mathrm{cm}}^{-1}$)	Non-scattering CNR	Scattering CNR
49.6	1.243	18.9	13.1
24.7	0.619	9.0	5.6
10.3	0.258	3.7	2.6
4.9	0.122	1.3	0.8
1.9	0.048	0.6	0.3

### 3.3 Multispectral Imaging

**Fig. 7:**
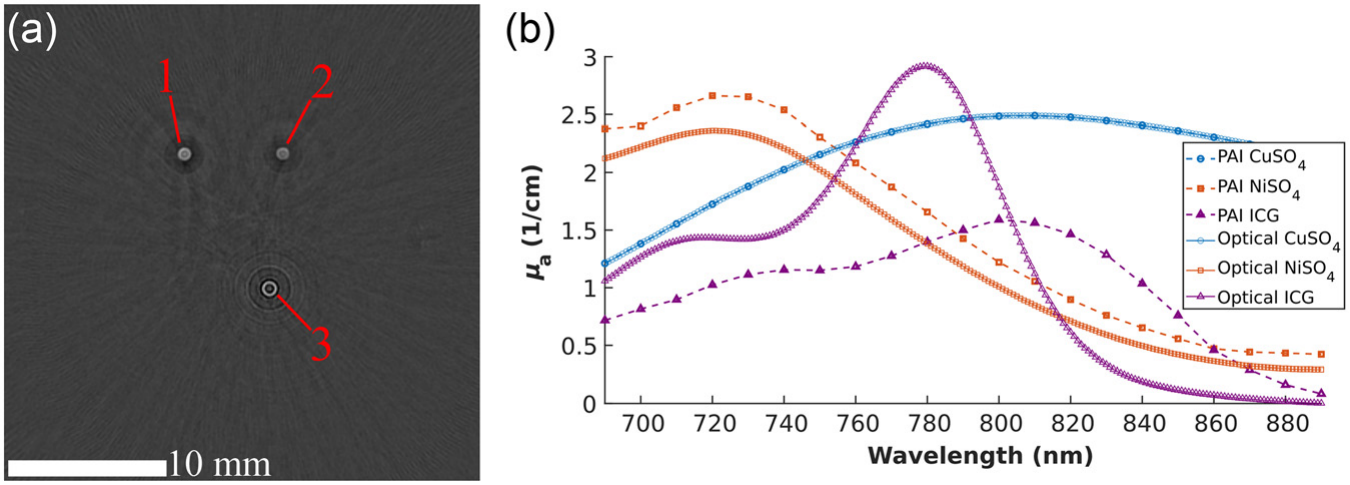
(a) A transverse slice of the multispectral phantom PAT volume excited at 780 nm. The three samples are visible: ${\mathrm{CuSO}}_{4}$ (1), ${\mathrm{NiSO}}_{4}$ (2), and ICG (3). (b) The PA spectrum of the samples, normalized to the ${\mathrm{CuSO}}_{4}$ optical spectrum.

A representative transverse slice of the multispectral phantom excited at 780 nm is shown in Fig. 7(a). In the sample slice, all three PAI contrast agents are visible: ${\mathrm{CuSO}}_{4}$ (1), ${\mathrm{NiSO}}_{4}$ (2), and ICG (3). The sulfates are visualized as more uniform circles than the ICG sample, which has a ring shape because of ICG’s affinity to adsorb to the PTFE wall of the microcuvette.^45^

The measured PA spectra of the contrast agents normalized to the optical absorption spectrum of the copper sulfate sample are shown in Fig. 7(b). The ${\mathrm{NiSO}}_{4}$ sample’s PA spectrum matches its optical spectrum in profile and closely in relative amplitude, which is an expected result from a PA stable sulfate.^46^ The ICG sample has a significantly different PA spectrum compared to its optical spectrum, in both profile and amplitude. The difference in absorption peaks of 720 and 780 nm in its optical spectrum is diminished in the PA spectrum, where the two peaks have a smaller difference relative to the ICG spectrum’s highest peak. The PA absorption spectrum is also redshifted by 20 nm with respect to the optical absorption spectrum. Both of these departures of the PA spectrum from the optical spectrum of ICG are known results from others’ previous work on characterizing its aggregation state.^47^ In this study, however, the differences in ICG’s PA and optical spectra can be attributed to the aggregation of the dye’s particles on the walls of the PTFE microcuvettes.^45^

### 3.4 Optical Spatial Resolution

The optical spatial resolution phantom is shown in Fig. 4, with the cases of the resolution chart outside of the imaging chamber [Fig. 4(a)], inside the imaging chamber without water [Fig. 4(b)], and inside the imaging chamber filled with water [Fig. 4(c)]. The final measured optical spatial resolution results are in Table 2. In air, the measured spatial resolution of the camera is consistent with the theoretical spatial resolution limit of 39  μm for the camera’s hardware (Sec. S6 in the Supplementary Material). In water, the spatial resolution of the camera is lower than air due to increased scattering and refraction, with the horizontal spatial resolution in water being significantly lower than the vertical resolution because of the imaging chamber’s cylindrical shape affecting the refraction angle of light in the horizontal directions.

**Table 2:** Optical spatial resolution limit of the TriTom in air and DI water.

Medium	Vertical resolution (μm)	Horizontal resolution (μm)
Air	50	50
DI water	70	112

### 3.5 Fluorescence Sensitivity

**Fig. 8:**
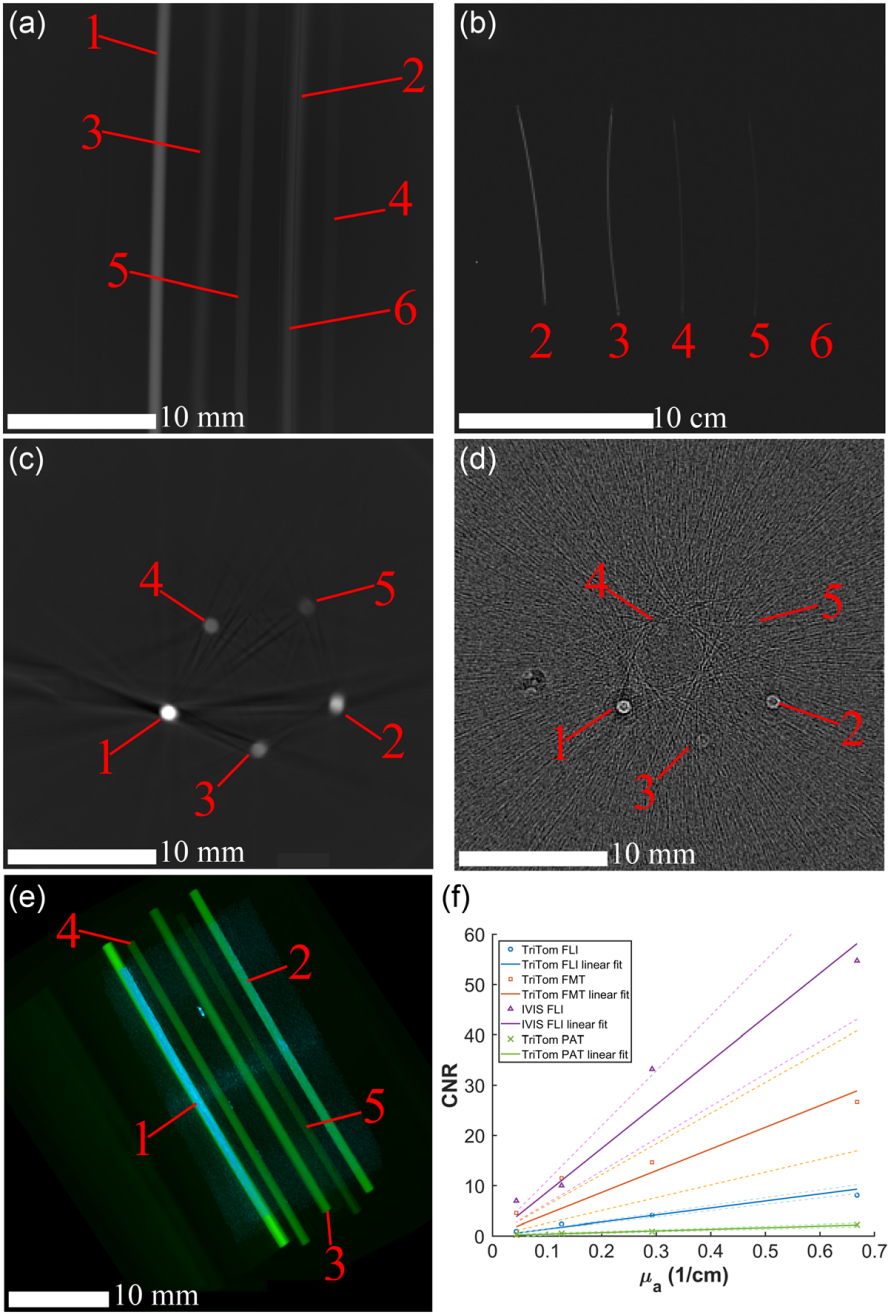
FL images of the FL sensitivity phantom imaged with the (a) TriTom and (b) IVIS Spectrum. A transverse plane of the FL sensitivity phantom’s (c) FMT volume and (d) PAT volume. (e) 3D MIP render of the FMT volume (green) and PAT volume (blue) superimposed. (f) CNR plot and linear fit of the IR-800 samples imaged with TriTom FLI, IVIS Spectrum FLI, TriTom FMT, and TriTom PAT. Identified are the samples of IR-800: 28.0  μM (1), 13.7  μM (2), 6.0  μM (3), 2.6  μM (4), 0.9  μM (5), and control water (6).

Figure 8 shows images of the FL sensitivity phantom imaged using TriTom’s PA and FL modalities, as well as IVIS Spectrum FLI. All samples of IR-800 are detected in both the TriTom and IVIS Spectrum FL images [Figs. 8(a) and 8(b)]. The absence of the highest sample concentration in the IVIS Spectrum FLI was to capture a better dynamic range of the whole image. A representative transverse slices of the TriTom’s FMT volume, reconstructed from the 360 FLI images, and the TriTom PAT volume are shown in Figs. 8(c) and 8(d), respectively. While all five samples are visible in both images, the two lowest concentration samples are barely detectable above the noise background in the PAT image. The CNR comparison plot [Fig. 8(f)] confirms that the FMT sensitivity of the TriTom is superior to the PAT sensitivity, with all samples of the FMT volume having a higher CNR than its PAT counterpart. This result, combined with the fact that IR-800 (quantum yield = 9%)^48^ favors PA compared to other fluorescent agents, that displays the strength of FLI as a companion modality to PAI through its enhanced molecular sensitivity.

Compared to the IVIS Spectrum FLI image, the TriTom’s FL CNR is significantly lower for the imaged IR-800 samples. A circular high background noise pattern is detected in the TriTom’s FL images, which is thought to be caused by light leakage of the excitation pulse through the optical filter from light at large incident angles. This high background noise, which propagates to the FMT volume as well, drastically decreases the reported CNR of the TriTom’s FL modality due to an increased background standard deviation.

### 3.6 Imaging *in Vivo*

**Fig. 9:**
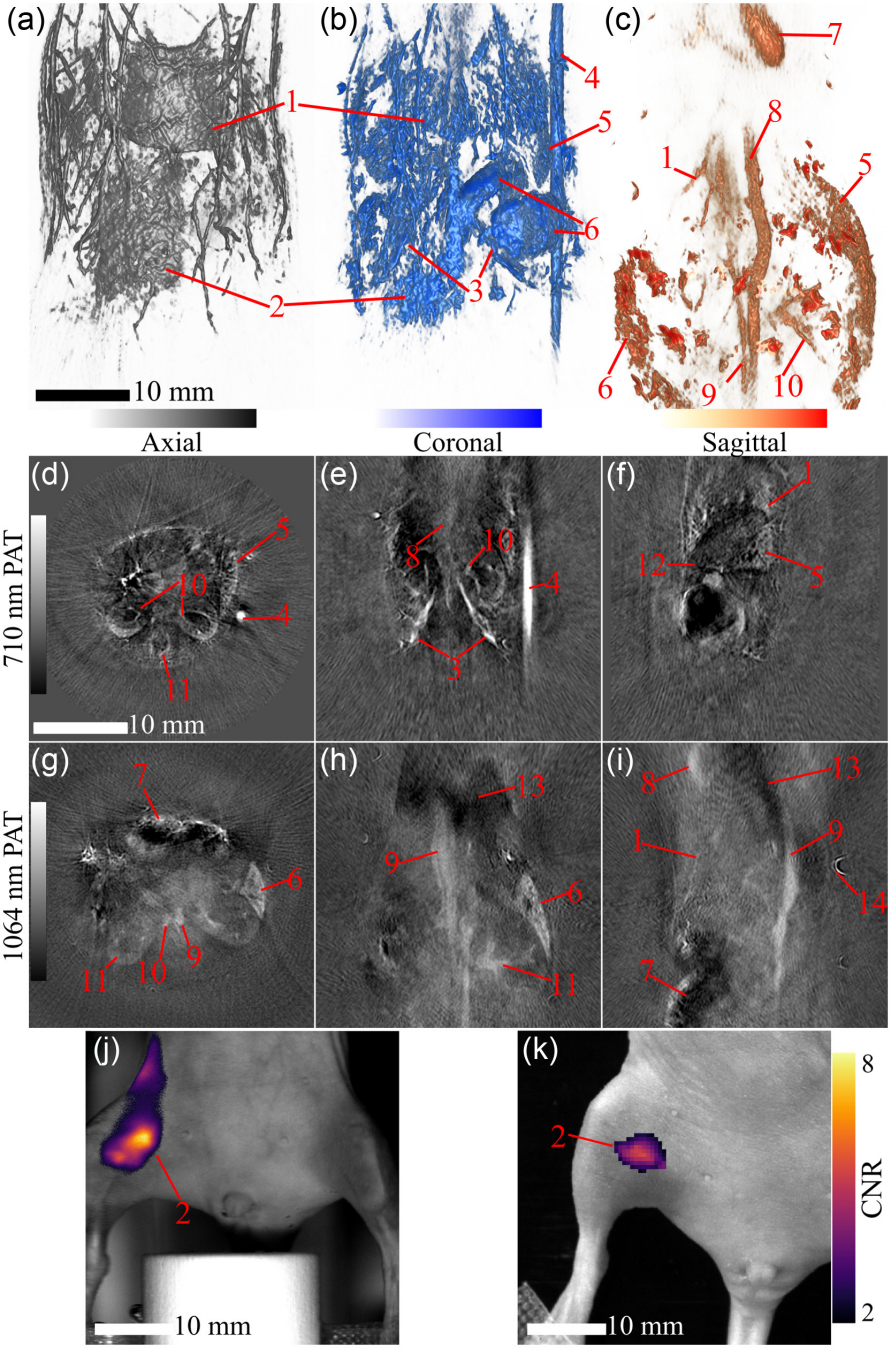
PAT volumes and slices of *in vivo* mouse scans. The top PAT row shows 3D renders of the PAT volumes scanned at wavelengths (a) 532 nm for superficial structures, (b) 710 nm for deep structures with deoxygenated hemoglobin, and (c) 1064 nm for deep structures with oxygenated hemoglobin in the coronal (ventral) view. The center PAT row has three slices of interest from the 710 nm volume: (d) axial, (e) coronal, and (f) sagittal. The bottom PAT row also has three slices from the 1064-nm volume, with the same view orientations of (g) axial, (h) coronal, and (i) sagittal. The FLI row has FL CNR>2 maps overlaid on photographs using a TriTom (j) and IVIS Lumina II (k), the color bar is normalized for both instruments. Key anatomical structures and a fiducial are labeled: liver/liver vessels (1), injection site (2), iliac vessels (3), copper sulfate fiducial (4), spleen (5), intestines (6), heart (7), aorta (8), vena cava (9), kidney/kidney vessels (10), vertebra (11), pancreas (12), lungs (13), and hair follicle (14). Scalebars on the 3D (a, b, c), and 2D (d, e, f, g, h, i, j, and k) images are 10 mm. Photoacoustic image color bars are normalized from 0 to 1 from arbitrary units; the FL map color bar represents the CNR of the fluorescent image.

To evaluate the *in vivo* PAT imaging capabilities of the TriTom, PAI scans with three excitation wavelengths were acquired in two mice. Figures 9(a) and 9(b) show 3D volume renders of the 532 and 710 nm scans of the first mouse following administration of GC-AuNPs mixed with ICG. The 532-nm PAT volume shows high-resolution images of superficial vasculature in the abdominal region of the mouse, because Hb and ${\mathrm{HbO}}_{2}$ are highly absorbent at 532 nm. Additionally, high intensity targets at the liver and injection site can be observed indicating accumulation of the GC-AuNPs which have an optical absorption peak near 532 nm. The 710-nm excitation volume provides deeper tissue information, due to improved light penetration, of Hb in internal anatomical structures such as the iliac blood vessels. A single PAI scan at 1064-nm excitation was acquired in the thoracic region of the third mouse [Fig. 9(c)]. At this wavelength, deep ${\mathrm{HbO}}_{2}$-rich structures are visible including the heart, aorta, vena cava, and kidney vasculature. Figure 9(d)–9(i) show select 2D slices from the 710 and 1064 nm volumes with labeled anatomy and targets that are not apparent in the 3D renderings. These images demonstrate that the TriTom provides high-resolution PAT images of both superficial and deep tissue structures with and without an exogenous contrast agent.

Comparative FLI scans were captured for a mouse using the TriTom’s FL mode and an IVIS Lumina II. 24-hr after the GC-AuNP mixed with ICG injection, the mouse was imaged in a IVIS Lumina II first, then using a TriTom. CNR maps were calculated to directly compare the images acquired with the two systems. The fluorescent image captured using the TriTom [Fig. 9(j)] detects a high intensity target at the injection site (2), consistent with the reference IVIS Lumina II [Fig. 9(k)] image. The TriTom image depicts the contrast agent diffusing from the injection site towards just above the mouse’s right upper hind limb, whereas the IVIS Lumina II image shows the contrast agent localized to the injection site.

## 4 Discussion

This work introduces the TriTom, a preclinical imaging system that integrates 3D PA and FL tomography imaging in a single instrument. We describe the characteristics of the TriTom and determine the spatial resolution and sensitivity limits of the system’s imaging modalities through phantom studies. Further, we demonstrate the ability of the TriTom to generate whole-body *in vivo* images in small animal models.

The spatial resolution of the TriTom’s PA mode was determined by evaluating transverse (radial and tangential) and longitudinal line profiles of a spatial resolution phantom volume. We calculated the average transverse PA spatial resolution as 173±17  μm (from a range of 142 to 215  μm) and the longitudinal resolution as 640±120  μm (from a range of 487 to 846  μm). There is a noticeable difference between the determined transverse and longitudinal PA spatial resolutions, where the longitudinal result is over 3× the transverse estimate and has a substantial uncertainty. Two factors contribute to this: the dual-peak profile of the horizontal monofilaments and the light beam profile at the phantom. Likely a result of not accounting for the PA detector’s manufacturing tolerances in the reconstruction, the horizontal monofilaments are visualized as a dual-peak target, as if the probe was not perfectly focused for targets along its centerline radius. Further calibrating the reconstruction algorithm to account for the PA detector’s radius and arc length tolerances would result in a more coherent horizontal monofilament image. The discrepancy between the FWHM of the two measured horizontal monofilaments is caused by an uneven vertical illumination pattern at the phantom. The light distribution of the fiber bundles used in this experiment was known to be disproportionately skewed toward the top of the light bar. The discrepancy in FWHM measurements would likely be minimized by optimizing the fiber bundles to evenly illuminate the phantom.

A standard optical spatial resolution phantom, a USAF 1951 test chart, was used to measure the spatial resolution of the camera and optical components that make up the TriTom’s FL mode. Expectedly, the optical spatial resolution was superior when the TriTom was filled with air instead of water. Although the optical spatial resolution is presented, a more fitting measurement would be with a fluorescent test chart to determine the fluorescent spatial resolution of the system. The optical spatial resolution measurement result is far more precise, on the order of 100  μm, than conventional FLI spatial resolution, on the order of millimeters at depth,^30^ and therefore shows that the FL mode of the system is capable of spatially quantitative measurements in the context of *in vivo* FLI.

The sensitivity limit of the TriTom’s PA modality was evaluated by scanning a phantom constructed from microcuvettes filled with copper sulfate in decreasing concentrations. In addition to its optical absorption peak in the NIR window (810 nm), the concentration of copper sulfate has a linear effect on the intensity of measured PA signals.^35^ This relationship between concentration and PA amplitude was affirmed in our measurements, where we observed a positive linear correlation between the concentration of copper sulfate and the amplitude of the measured PA signal for both the scattering and non-scattering phantom environments. These findings demonstrate the ability of the TriTom’s PA mode to accurately detect relative sample concentrations in vitro. While the sample concentrations we used determined that the scattering and non-scattering LLOD had the same range, the scattering CNR values were at least 30% lower than the non-scattering CNR. Using more discrete sample concentrations of copper sulfate in this range would help to determine the exact LLOD of the TriTom. Because the PA sensitivity is also a factor of target size with respect to a detector’s center frequency and bandwidth,^49^ using variable sizes of microcuvettes would be a new criteria to further determine the LLOD. Additional factors, such as spectral unmixing, improved filtering of the reconstruction algorithm, and the use of an alternate coupling medium would also have significant effects on the reported PA sensitivity.

The sensitivity of the TriTom’s FL and PA modes were also characterized using an FL phantom consisting of several concentrations of a dual PA and FL contrast agent. While the TriTom FMT sensitivity was found to be superior to PAT, FL images acquired with the IVIS Spectrum had higher CNR values for all samples due to the high noise background in the TriTom FL images. By improving the light tightness of the system’s FL mode and optimizing the selection of the optical emission filters, the variance in the noise background will be reduced, increasing the sensitivity of FL images acquired with the TriTom.

To evaluate the multispectral imaging capabilities of the TriTom, the PA spectra of several PA contrast agents were compared to their measured optical attenuation spectra. The PA spectrum of the nickel sulfate sample showed a well-matched profile to its optical attenuation spectrum. This result was expected because the sample is known to be PA stable (resistance to photobleaching).^35^ However, the PA spectrum profile of ICG did not match its optical absorption spectrum, having a different shape and an overall redshift of 20 nm caused by the aggregation of ICG particles on the wall of the PTFE microcuvette. These characteristics of ICG and the effect on the measured PA spectrum are consistent with previously reported literature.^47^

In addition to characterizing the PAT and FMT modalities of the system, we acquired *in vivo* images of three mice to demonstrate the potential biomedical applications of the TriTom. The reconstructed 1064-nm PAT volume shows high-resolution images of ${\mathrm{HbO}}_{2}$-rich anatomical structures. More specifically, the high energy scan acquired at this wavelength with the TriTom enabled visualization of both superficial and deep tissues containing ${\mathrm{HbO}}_{2}$, including the aorta, kidneys, and liver vasculature. These structures are typically unable to be differentiated from Hb-derived PA signals without the use of advanced techniques such as spectral unmixing.

To demonstrate TriTom imaging with an exogenous contrast agent, we evaluated the local clearance of a subcutaneously injected GC-AuNP and ICG mixture. An FL CNR map with the TriTom shows a high-intensity target at the injection site and above the right hind limb, 24-hr postinjection. This strong FL signal was also observed in the IVIS Lumina II FL images, confirming the *in vivo* FL imaging capabilities of the TriTom. While the TriTom has higher CNR values at the injection site than the IVIS Lumina II, it also has a higher noise floor, as can be seen by the presence of the noise artifacts at the base of the mouse’s tail and the bottom border of the image (Fig. S5 in the Supplementary Material). Along with the previously mentioned ways to improve the FLI capabilities of the TriTom, the case of an *in vivo* FL scan could be significantly improved by relocating the orthogonal excitation lightbars to be epi-excitation ports with respect to the camera detector.

Photoacoustic scans were also acquired at two excitation wavelengths 6 hr after the mixture was injected in a separate animal. The PAT volume reconstructed from the 532-nm laser excitation scan shows a high-intensity PA target at the injection site and the liver, consistent with the 710-nm excitation PAT volume. Because these excitation wavelengths correspond to the optical absorption peaks of the contrast agent mixture, these PA signals indicate that portions of the mixture remained at the injection site while the rest drained into the liver. Combined, the PAT and FL images demonstrate that the TriTom can detect the accumulation and clearances of exogenous contrast agents.

Given the complementary nature of PA and FL imaging, prototype imaging systems that integrate these technologies have been previously investigated.^31^^–^^33^ While these imaging systems highlight the advantages of combining PA and FL imaging, they are limited to providing individual 2D images of the subject. The TriTom, however, is uniquely designed to simultaneously collect volumetric PA (azimuthal frames) and FL (multiangle planar frames) data with a temporal resolution limited only by the maximum speed of the rotational motor. As a result, the system provides 3D whole-body snapshots of the image target that better capture systemic processes than 2D slices. Future work will include an optimized light excitation to accommodate increased rotational speeds and a dynamic image reconstruction algorithm, both of which should improve the system’s temporal resolution and imaging capabilities.

## 5 Conclusion

We introduced a preclinical multimodality PA and FL imaging platform and characterized its spatial resolution and sensitivity. Several limitations of the system’s subcomponents are addressed (e.g., longitudinal spatial resolution, FLI background noise, etc.), and improvements to the system technology are identified for future development. Through the demonstration of *in vivo* PAI mouse scans, we establish that the 3D scanning TriTom imaging system can be used for practical biomedical applications in small animal models. The imaging system detailed has the potential for many implementations in preclinical studies that can impact the applications of cancer therapeutics,^50^ stem cells,^51^ developmental biology,^52^ functional assessment, and the development of contrast agents.^45^

## Code, Data, and Materials Availability

The code, data, and materials presented in this paper are not publicly available. Requests for the code and data will be considered if received by the author.
