# Supplementary material for: Characterizing a photoacoustic and fluorescence imaging platform for preclinical murine longitudinal studies
Source: J Biomed Opt. 2023 Mar 7;28(3):036001. doi: 10.1117/1.JBO.28.3.036001 (PMC9990133; doi:10.1117/1.JBO.28.3.036001)
Supplement: Supplementary file 1 [file JBO_028_036001_SD001.pdf]

Supplemental Materials for Characterizing a Photoacoustic and Fluorescence Imaging Platform for Pre-clinical Murine Longitudinal Studies

S1 Optical Spatial Resolution

Figure S1 shows the extracted line profiles from the optical spatial resolution test chart's smallest resolvable element.

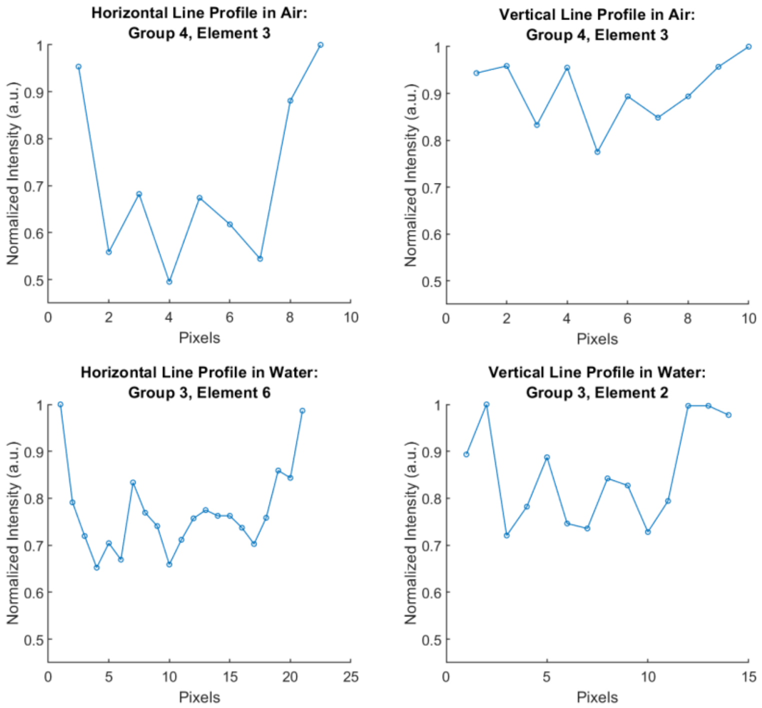

Figure S1: Line profiles from the least resolvable group and element number of the optical spatial resolution phantom. The three troughs in the profiles indicate that the three bars of the USAF 1951 resolution test chart's elements are distinguishable and therefore resolvable.

S2 In Vivo Mouse Scan

An example photograph of a live mouse mounted in the TriTom imaging chamber is shown in Fig. S2.

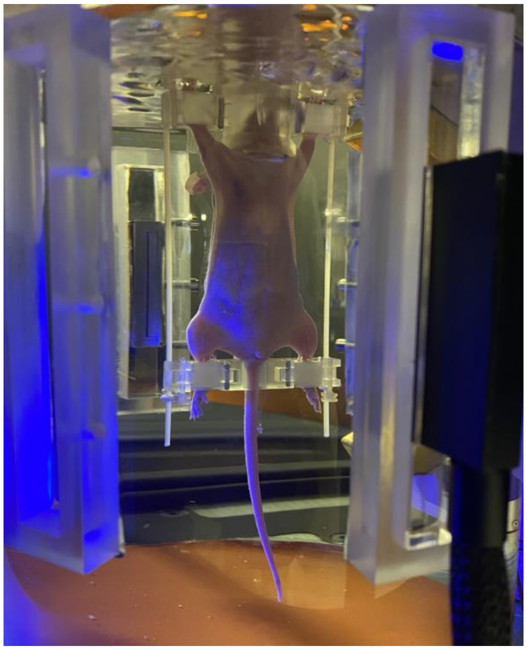

Figure S2: Photo of a live mouse inside the TriTom imaging chamber during an *in vivo* imaging experiment. The anesthetized animal is secured to the mouse restrainer via cushioned paw mounts for the front and hind limbs. The mouse's nose and mouth are positioned inside the restrainer nose cone through which a continuous supply of anesthesia gas is delivered. The flow rate of the anesthesia gas is optimized to maintain a stable bubble surrounding the nose and mouth while the animal is submerged.

S3 Contrast Agent Optical Absorption Spectrum

The contrast agent injected into the mice in this study was a mixture of glycol-chitosan-coated gold nanospheres (GC-AuNPs) mixed with indocyanine green (ICG). The optical absorption spectrum of the contrast agent mixture is shown in Fig. S3.

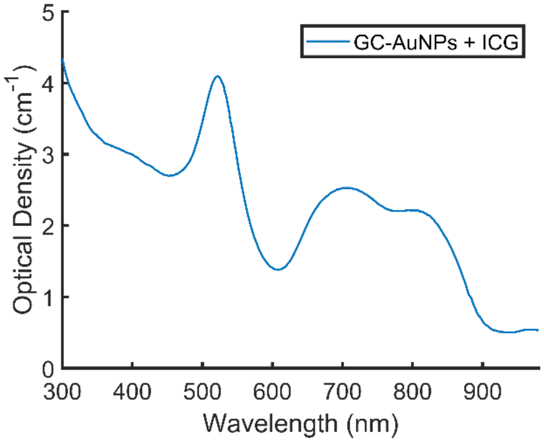

Figure S3: Optical absorption spectrum of glycol-chitosan-coated gold nanospheres (GC-AuNPs) mixed with indocyanine green (ICG). This dye was subcutaneously injected into a mouse's right mammary fat pad to track lymphatic drainage. The animal was then imaged with the TriTom.

S4 Fluorescence Imaging In Vivo Segmentation

To calculate the contrast-to-noise ratio (CNR) of an image, a target area and background area must be designated. In this case, the target area was chosen as the injection site in a fluorescent image and the background area was chosen as the area of a mouse's body (Fig. S4).

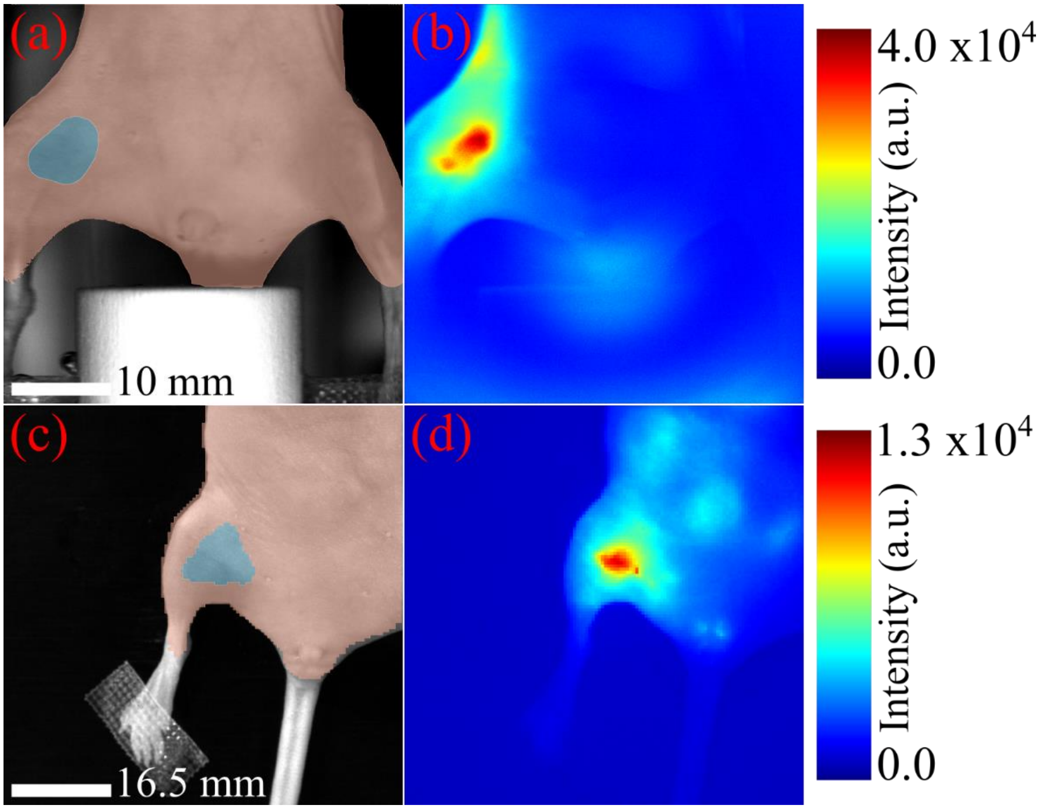

Figure S4: Photographs of a mouse captured using a TriTom (a) and IVIS Lumina II (c) with overlayed regions-of-interest (ROI) segmentations. The blue ROI was selected to include the high intensity fluorescence from the injection site; the orange ROI was selected to encompass the mouse's body, excluding the injection site's fluorescence ROI and background not included in the mouse's body. The raw fluorescence images of the TriTom (b) and IVIS Lumina II (d) are displayed in their absolute scale (16-bit).

S5 Fluorescence CNR Map Visualization

Masking these chosen areas as segmentations over the mouse's body, CNR maps can be calculated for the *in vivo* TriTom and IVIS Lumina II fluorescent images, allowing for a direct comparison of the two imaging systems' results (Fig. S5).

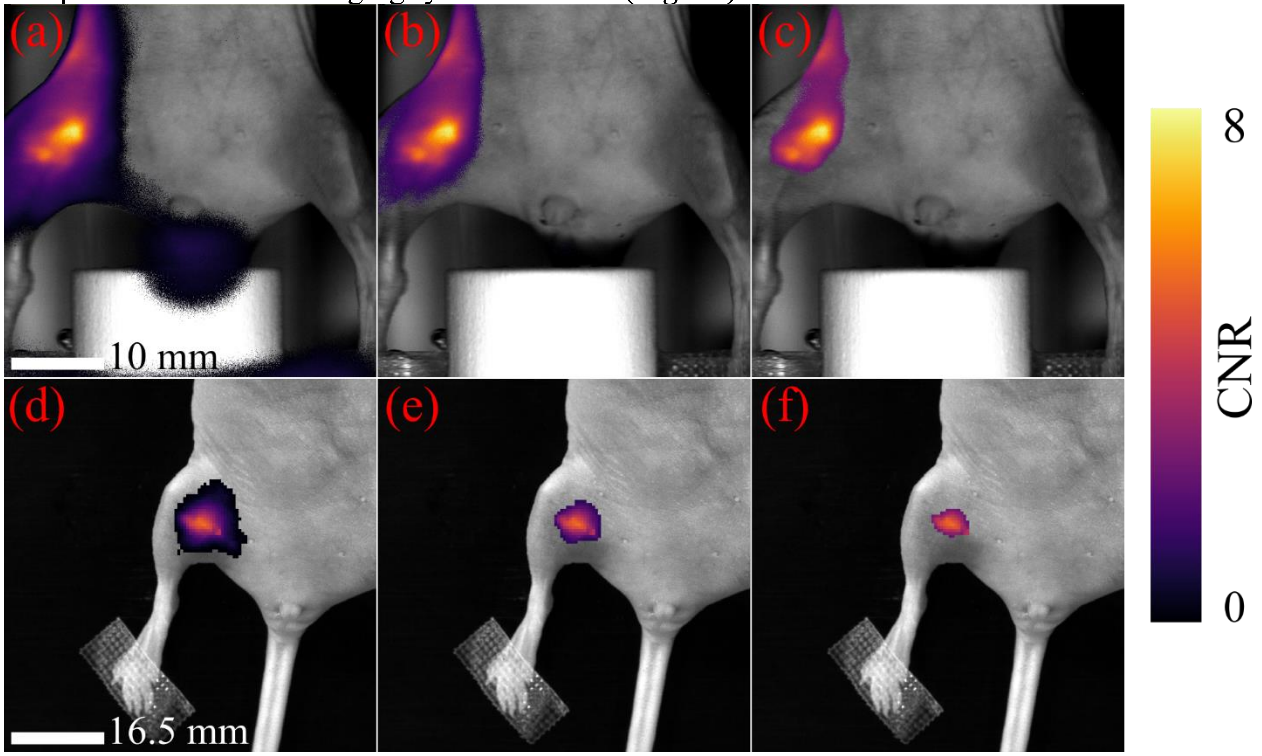

Figure S5: Contrast-to-noise ratio (CNR) maps of the fluorescence images, using the inclusion and background segmentations shown in (Fig. S4), overlayed on photographs of TriTom images (a, b, c) in the top row and IVIS Lumina II images (d, e, f) in the bottom row. The left column shows the CNR > 0 fluorescence map, middle column shows the CNR > 1 fluorescence map, right column shows the CNR > 2 fluorescence map. The significance being, using the definition of CNR in (Eq. 1), CNR > 0 is the fluorescence detected including background noise, CNR > 1 is the fluorescence detected at one standard deviation from the background noise, and CNR > 2 is the fluorescence map that meets the minimum criterion for target detection.

S6 Spatial Resolution Limit of Optical Detector

The spatial resolution limit of the optical detector is calculated below, with relevant parameters listed. Our optical detection channel has a final field-of-view at the TriTom's rotational axis of 40 x 40 mm^2. This calculation gives the theoretical limit of optical spatial resolution detection based on the hardware used in the TriTom, namely the camera and objective.

Tucsen Dhyana 400D parameters:

Pixel Size (s): 6.5 µm x 6.5 µm

Number of Pixels (p): 2048 x 2040

Image Field of View (FoV): 40 mm

Limiting sensor resolution:

$$R_I = \frac{1}{2 \times s} = \frac{1}{2 \times 6.5\ \mu m} \times \frac{1000\ \mu m}{1\ mm} = 76.92\ \frac{lp}{mm}$$

Where R_I is the image space resolution.

Sensor dimensions:

$$S_H = s\ \times p = 6.5\ \mu m\ \times 2048\ \times \frac{1\ mm}{1000\ \mu m} = 13.31\ mm$$

$$S_V = \ 6.5\ \mu m \times\ 2040 \times \frac{1\ mm}{1000\ \mu m} = 13.26\ mm$$

Where subscript H stands for horizontal and subscript V stands for vertical.

Magnification:

$$m = \frac{\max\ (S_H, S_V)}{FoV} = \frac{13.31\ mm}{40\ mm} = 0.33X$$

Spatial resolution limit:

$$R\ = R_I \times m = 76.92\frac{lp}{mm} \times 0.33 = 25.38\frac{lp}{mm} = 39.4\ \mu m$$

Where R is the optical spatial resolution limit of the system.
